# Pyuria or Pus in the Urine and Its Treatment

**Published:** 1885-03

**Authors:** 


**Pyuria or Pus in the Urine and its Treatment.** Comprising the Diagnosis and Treatment of Acute and Chronic Urethritis, Prostatitis, Cystitis and Pyelitis, with Special Reference to their Local Treatment. By DR. ROBERT ULTZMANN, Professor of Genito-Urinary Diseases in the Vienna Polyclinic. Translated by permission by WALTER B. PLATT, M. D. New York: D. Appleton & Co. 1884.

The title page, as above, sufficiently explains the scope of this work of about one hundred pages. That it is by Ultzmann is evidence that the subject is thoroughly treated. Dr. Platt is to be complimented on the success of his translation.

